# 4-Chloro-*N*-(2-chloro­phen­yl)benzamide

**DOI:** 10.1107/S1600536808028882

**Published:** 2008-09-13

**Authors:** Aamer Saeed, Rasheed Ahmad Khera, Kazuma Gotoh, Hiroyuki Ishida

**Affiliations:** a Department of Chemistry, Quaid-I-Azam University, Islamabad 45320, Pakistan; b Department of Chemistry, Faculty of Science, Okayama University, Okayama 700-8530, Japan

## Abstract

In the mol­ecular structure of the title compound, C_13_H_9_Cl_2_NO, the amide N—C=O plane makes dihedral angles of 31.53 (8) and 36.23 (8)°, respectively, with the 4-chloro- and 2-chloro­phenyl rings. The dihedral angle between the two benzene rings is 6.25 (8)°. The mol­ecules are stacked in columns along the *b* axis through inter­molecular N—H⋯O hydrogen bonds. The columns are further connected by weak C—H⋯O hydrogen bonds. The compound is not isomorphous with the fluoro analogue.

## Related literature

For general background, see: Capdeville *et al.* (2002 ▶); Chopra & Row (2005 ▶); Ho *et al.* (2002 ▶); Igawa *et al.* (1999 ▶); Jackson *et al.* (1994 ▶); Makino *et al.* (2003 ▶); Zhichkin *et al.* (2007 ▶). For related structures, see: Chopra & Row (2005 ▶).
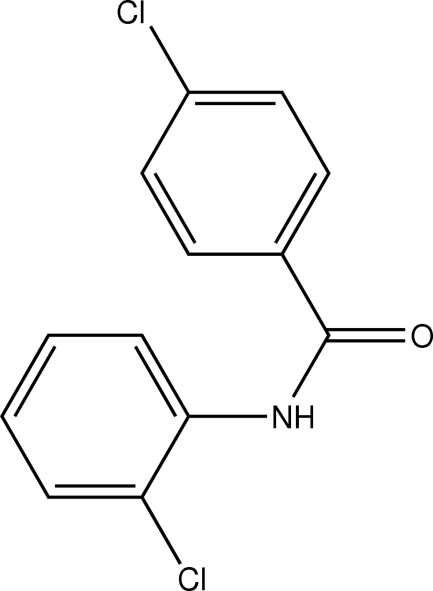


## Experimental

### 

#### Crystal data

C_13_H_9_Cl_2_NO
*M* *_r_* = 266.13
Monoclinic, 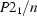

*a* = 10.7913 (14) Å
*b* = 4.8078 (6) Å
*c* = 23.570 (3) Å
β = 97.718 (3)°
*V* = 1211.8 (3) Å^3^
*Z* = 4
Mo- *K*α radiation
μ = 0.52 mm^−1^
*T* = 223 (1) K
0.35 × 0.31 × 0.05 mm

#### Data collection

Rigaku R-AXIS RAPID II diffractometer
Absorption correction: multi-scan (*ABSCOR*; Higashi, 1995 ▶) *T*_min_ = 0.884, *T*_max_ = 0.975
14924 measured reflections
3527 independent reflections
1847 reflections with *I* > 2σ(*I*)
*R*_int_ = 0.042

#### Refinement

*R*[*F* ^2^ > 2σ(*F* ^2^)] = 0.051
*wR*(*F* ^2^) = 0.154
*S* = 1.00
3527 reflections
158 parameters
H atoms treated by a mixture of independent and constrained refinement
Δρ_max_ = 0.23 e Å^−3^
Δρ_min_ = −0.39 e Å^−3^

### 

Data collection: *PROCESS-AUTO* (Rigaku/MSC, 2004 ▶); cell refinement: *PROCESS-AUTO*; data reduction: *CrystalStructure* (Rigaku/MSC, 2004 ▶); program(s) used to solve structure: *SHELXS97* (Sheldrick, 2008 ▶); program(s) used to refine structure: *SHELXL97* (Sheldrick, 2008 ▶); molecular graphics: *ORTEP-3* (Farrugia, 1997 ▶); software used to prepare material for publication: *CrystalStructure* and *PLATON* (Spek, 2003 ▶).

## Supplementary Material

Crystal structure: contains datablocks global, I. DOI: 10.1107/S1600536808028882/bh2189sup1.cif

Structure factors: contains datablocks I. DOI: 10.1107/S1600536808028882/bh2189Isup2.hkl

Additional supplementary materials:  crystallographic information; 3D view; checkCIF report

## Figures and Tables

**Table 1:** Hydrogen-bond geometry (Å, °)

*D*—H⋯*A*	*D*—H	H⋯*A*	*D*⋯*A*	*D*—H⋯*A*
N1—H1⋯O1^i^	0.85 (2)	2.12 (2)	2.901 (2)	154 (2)
C2—H2⋯O1^ii^	0.94	2.59	3.456 (3)	153

Symmetry codes: (i) 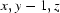; (ii) 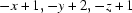.

**Fig. 1.**
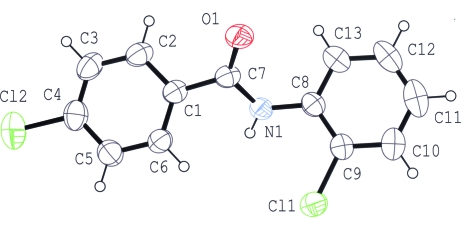
The molecular structure of the title compound. The displacement ellipsoids are drawn at the 40% probability level.

**Fig. 2.**
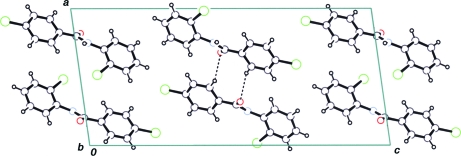
Crystal packing, viewed along the b axis. Intermolecular C—H···O hydrogen bonds are shown as dashed lines.

## supplementary crystallographic information

### Comment

The benzanilide core is present in compounds with a wide range of biological activities, and for this reason it has been called a privileged structure. Benzanilides serve as intermediates towards benzothiadiazin-4-ones (Makino *et al.*, 2003), benzodiazepine-2,5-diones (Ho *et al.*, 2002), and 2,3-disubstituted 3*H*-quinazoline-4-ones (Zhichkin *et al.*, 2007). Benzanilides have established their efficacy as centroid elements of ligands that bind to a wide variety of receptor types. Thus, benzanilides containing aminoalkyl groups originally designed as peptidomimetic compounds, have been incorporated in an Arg-Gly-Asp cyclic peptide, yielding a high affinity GPIIb/IIIa ligand (Jackson *et al.*, 1994). Imatinib is an ATP-site binding kinase inhibitor and platelet-derived growth factor receptor kinase (Capdeville *et al.*, 2002). Benzamides have activities as acetyl-CoA carboxylase and farnesyl transferase inhibitors (Igawa *et al.*, 1999).

In the crystal structure of the title compound (Fig. 1), the molecules are stacked in columns along the *b* cell-axis through intermolecular N—H···O hydrogen bonds (Table 1). The columns are also connected by weak C—H···O hydrogen bonds (Fig. 2). No significant π–π interactions are observed in the columns. The title compound is not isomorphous with the F analogue compound, 4-fluoro-*N*-(2-fluorophenyl)-benzamide, which exhibits a dimorphic behaviour, with non-centrosymmetric space groups *P*2_1_ and *Pca*2_1_ (Chopra & Row, 2005). The different crystal structures of the F analogue are probably originated from the intermolecular C—H···F interactions.

### Experimental

4-Chorobenzoyl chloride (5.4 mmol) in CHCl_3_ was treated with 2-chloroaniline (21.6 mmol) under a nitrogen atmosphere at reflux for 4 h. Upon cooling, the reaction mixture was diluted with CHCl_3_ and washed consecutively with aq. 1 *M* HCl and saturated aq. NaHCO_3_. The organic layer was dried over anhydrous sodium sulfate and concentrated under reduced pressure. Crystallization of the residue in CHCl_3_ afforded the title compound (84%). Anal. calcd. for C_13_H_9_Cl_2_NO: C 58.67, H 3.41, N 5.26%; found: C 58.23, H 3.46, N 5.08%.

### Refinement

The N-bound H atom was located in a difference map and refined freely. Other H atoms were positioned geometrically (C—H = 0.94 Å) and treated as riding atoms, with *U*_iso_(H) = 1.2*U*_eq_(C).

### Crystal data

**Table:**

C_13_H_9_Cl_2_NO	*F*(000) = 544.00
*M**_r_* = 266.13	*D*_x_ = 1.459 Mg m^−^^3^
Monoclinic, *P*2_1_/*n*	Mo *K*α radiation, λ = 0.71075 Å
Hall symbol: -P 2yn	Cell parameters from 8924 reflections
*a* = 10.7913 (14) Å	θ = 3.0–30.0°
*b* = 4.8078 (6) Å	µ = 0.52 mm^−^^1^
*c* = 23.570 (3) Å	*T* = 223 K
β = 97.718 (3)°	Plate, colourless
*V* = 1211.8 (3) Å^3^	0.35 × 0.31 × 0.05 mm
*Z* = 4	

### Data collection

**Table:**

Rigaku R-AXIS RAPID II diffractometer	1847 reflections with *I* > 2σ(*I*)
Detector resolution: 10.00 pixels mm^-1^	*R*_int_ = 0.042
ω scans	θ_max_ = 30.0°
Absorption correction: multi-scan (ABSCOR; Higashi, 1995)	*h* = −15→15
*T*_min_ = 0.884, *T*_max_ = 0.975	*k* = −6→6
14924 measured reflections	*l* = −31→33
3527 independent reflections	

### Refinement

**Table:**

Refinement on *F*^2^	0 restraints
Least-squares matrix: full	0 constraints
*R*[*F*^2^ > 2σ(*F*^2^)] = 0.051	H atoms treated by a mixture of independent and constrained refinement
*wR*(*F*^2^) = 0.154	*w* = 1/[σ^2^(*F*_o_^2^) + (0.079*P*)^2^] where *P* = (*F*_o_^2^ + 2*F*_c_^2^)/3
*S* = 1.00	(Δ/σ)_max_ < 0.001
3527 reflections	Δρ_max_ = 0.23 e Å^−^^3^
158 parameters	Δρ_min_ = −0.39 e Å^−^^3^

### Fractional atomic coordinates and isotropic or equivalent isotropic displacement parameters (Å^2^)

**Table:**

	*x*	*y*	*z*	*U*_iso_*/*U*_eq_	
Cl1	0.01681 (5)	0.18996 (11)	0.55784 (3)	0.0701 (2)	
Cl2	0.40856 (8)	0.29994 (18)	0.27592 (3)	0.1044 (3)	
O1	0.31900 (14)	0.9176 (3)	0.52035 (6)	0.0704 (4)	
N1	0.24949 (15)	0.4883 (3)	0.54060 (7)	0.0533 (4)	
H1	0.246 (2)	0.321 (5)	0.5293 (10)	0.074 (7)*	
C1	0.32607 (17)	0.5666 (4)	0.45021 (8)	0.0523 (4)	
C2	0.4236 (2)	0.6900 (4)	0.42566 (10)	0.0650 (6)	
H2	0.4721	0.8303	0.4456	0.078*	
C3	0.4487 (2)	0.6070 (5)	0.37257 (11)	0.0725 (6)	
H3	0.5152	0.6882	0.3565	0.087*	
C4	0.3765 (2)	0.4053 (5)	0.34303 (10)	0.0695 (6)	
C5	0.2787 (2)	0.2815 (5)	0.36606 (10)	0.0680 (6)	
H5	0.2295	0.1444	0.3454	0.082*	
C6	0.25440 (19)	0.3623 (4)	0.41982 (9)	0.0599 (5)	
H6	0.1887	0.2780	0.4359	0.072*	
C7	0.29897 (17)	0.6734 (3)	0.50645 (9)	0.0528 (5)	
C8	0.20311 (18)	0.5546 (4)	0.59237 (8)	0.0531 (4)	
C9	0.09424 (19)	0.4263 (4)	0.60525 (8)	0.0554 (5)	
C10	0.0457 (2)	0.4875 (5)	0.65537 (9)	0.0727 (6)	
H10	−0.0267	0.3965	0.6639	0.087*	
C11	0.1041 (3)	0.6823 (6)	0.69252 (11)	0.0870 (8)	
H11	0.0701	0.7295	0.7259	0.104*	
C12	0.2115 (3)	0.8069 (5)	0.68088 (11)	0.0833 (8)	
H12	0.2517	0.9368	0.7069	0.100*	
C13	0.2623 (2)	0.7451 (4)	0.63118 (10)	0.0694 (6)	
H13	0.3366	0.8322	0.6239	0.083*	

### Atomic displacement parameters (Å^2^)

**Table:**

	*U*^11^	*U*^22^	*U*^33^	*U*^12^	*U*^13^	*U*^23^
Cl1	0.0638 (4)	0.0686 (4)	0.0792 (4)	−0.0099 (2)	0.0140 (3)	−0.0038 (3)
Cl2	0.1190 (6)	0.1252 (6)	0.0763 (5)	0.0111 (5)	0.0399 (4)	−0.0089 (4)
O1	0.0844 (10)	0.0411 (7)	0.0895 (11)	−0.0058 (7)	0.0258 (8)	−0.0078 (7)
N1	0.0588 (10)	0.0416 (8)	0.0610 (10)	−0.0015 (7)	0.0138 (7)	−0.0073 (7)
C1	0.0492 (11)	0.0427 (9)	0.0664 (12)	0.0048 (8)	0.0125 (9)	0.0010 (8)
C2	0.0556 (12)	0.0544 (11)	0.0883 (16)	−0.0030 (9)	0.0215 (11)	−0.0019 (10)
C3	0.0654 (14)	0.0695 (13)	0.0890 (16)	0.0007 (11)	0.0333 (12)	0.0051 (12)
C4	0.0710 (14)	0.0737 (14)	0.0667 (13)	0.0155 (11)	0.0193 (11)	0.0037 (11)
C5	0.0673 (14)	0.0718 (13)	0.0649 (14)	−0.0011 (11)	0.0085 (10)	−0.0083 (10)
C6	0.0560 (12)	0.0605 (11)	0.0647 (12)	−0.0051 (9)	0.0137 (9)	−0.0010 (9)
C7	0.0468 (10)	0.0411 (9)	0.0714 (12)	0.0028 (7)	0.0114 (9)	−0.0016 (8)
C8	0.0581 (11)	0.0459 (9)	0.0546 (11)	0.0081 (8)	0.0050 (8)	−0.0026 (8)
C9	0.0584 (12)	0.0542 (10)	0.0536 (10)	0.0086 (9)	0.0078 (9)	0.0019 (9)
C10	0.0785 (15)	0.0826 (14)	0.0596 (12)	0.0170 (12)	0.0185 (11)	0.0073 (12)
C11	0.111 (2)	0.0959 (19)	0.0554 (14)	0.0370 (17)	0.0142 (14)	−0.0010 (13)
C12	0.105 (2)	0.0804 (16)	0.0593 (14)	0.0151 (15)	−0.0073 (13)	−0.0202 (11)
C13	0.0756 (15)	0.0632 (12)	0.0665 (14)	0.0028 (11)	−0.0010 (11)	−0.0106 (10)

### Geometric parameters (Å, °)

**Table:**

Cl1—C9	1.729 (2)	C5—C6	1.384 (3)
Cl2—C4	1.739 (2)	C5—H5	0.9400
O1—C7	1.230 (2)	C6—H6	0.9400
N1—C7	1.357 (2)	C8—C13	1.388 (3)
N1—C8	1.416 (2)	C8—C9	1.396 (3)
N1—H1	0.84 (2)	C9—C10	1.387 (3)
C1—C6	1.388 (3)	C10—C11	1.376 (4)
C1—C2	1.399 (3)	C10—H10	0.9400
C1—C7	1.487 (3)	C11—C12	1.364 (4)
C2—C3	1.375 (3)	C11—H11	0.9400
C2—H2	0.9400	C12—C13	1.391 (3)
C3—C4	1.373 (3)	C12—H12	0.9400
C3—H3	0.9400	C13—H13	0.9400
C4—C5	1.384 (3)		
			
C7—N1—C8	125.20 (16)	O1—C7—N1	122.49 (18)
C7—N1—H1	116.3 (16)	O1—C7—C1	121.24 (16)
C8—N1—H1	118.4 (16)	N1—C7—C1	116.26 (16)
C6—C1—C2	118.98 (18)	C13—C8—C9	118.34 (19)
C6—C1—C7	122.80 (16)	C13—C8—N1	122.11 (19)
C2—C1—C7	118.10 (18)	C9—C8—N1	119.55 (17)
C3—C2—C1	120.3 (2)	C10—C9—C8	121.1 (2)
C3—C2—H2	119.8	C10—C9—Cl1	119.00 (17)
C1—C2—H2	119.8	C8—C9—Cl1	119.86 (15)
C4—C3—C2	119.9 (2)	C11—C10—C9	119.5 (2)
C4—C3—H3	120.0	C11—C10—H10	120.2
C2—C3—H3	120.0	C9—C10—H10	120.2
C3—C4—C5	121.0 (2)	C12—C11—C10	120.0 (2)
C3—C4—Cl2	119.89 (18)	C12—C11—H11	120.0
C5—C4—Cl2	119.1 (2)	C10—C11—H11	120.0
C6—C5—C4	119.1 (2)	C11—C12—C13	121.2 (2)
C6—C5—H5	120.4	C11—C12—H12	119.4
C4—C5—H5	120.4	C13—C12—H12	119.4
C5—C6—C1	120.66 (19)	C8—C13—C12	119.8 (2)
C5—C6—H6	119.7	C8—C13—H13	120.1
C1—C6—H6	119.7	C12—C13—H13	120.1
			
C6—C1—C2—C3	−0.8 (3)	C2—C1—C7—N1	−151.15 (18)
C7—C1—C2—C3	−177.11 (19)	C7—N1—C8—C13	−39.9 (3)
C1—C2—C3—C4	1.1 (3)	C7—N1—C8—C9	140.0 (2)
C2—C3—C4—C5	−0.5 (3)	C13—C8—C9—C10	0.3 (3)
C2—C3—C4—Cl2	−179.88 (18)	N1—C8—C9—C10	−179.58 (17)
C3—C4—C5—C6	−0.4 (3)	C13—C8—C9—Cl1	179.47 (15)
Cl2—C4—C5—C6	179.04 (17)	N1—C8—C9—Cl1	−0.4 (2)
C4—C5—C6—C1	0.6 (3)	C8—C9—C10—C11	1.4 (3)
C2—C1—C6—C5	0.0 (3)	Cl1—C9—C10—C11	−177.82 (17)
C7—C1—C6—C5	176.07 (19)	C9—C10—C11—C12	−2.2 (4)
C8—N1—C7—O1	7.0 (3)	C10—C11—C12—C13	1.4 (4)
C8—N1—C7—C1	−171.95 (16)	C9—C8—C13—C12	−1.1 (3)
C6—C1—C7—O1	−146.2 (2)	N1—C8—C13—C12	178.72 (19)
C2—C1—C7—O1	29.9 (3)	C11—C12—C13—C8	0.3 (4)
C6—C1—C7—N1	32.7 (3)		

### Hydrogen-bond geometry (Å, °)

**Table:**

*D*—H···*A*	*D*—H	H···*A*	*D*···*A*	*D*—H···*A*
N1—H1···O1^i^	0.85 (2)	2.12 (2)	2.901 (2)	154 (2)
C2—H2···O1^ii^	0.94	2.59	3.456 (3)	153
C13—H13···O1	0.94	2.46	2.884 (3)	108

Symmetry codes: (i) *x*, *y*−1, *z*; (ii) −*x*+1, −*y*+2, −*z*+1.
